# The cardiovascular effects of hyperoxia during and after cabg surgery

**DOI:** 10.1186/2197-425X-3-S1-A949

**Published:** 2015-10-01

**Authors:** B Smit, YM Smulders, MC de Waard, C Boer, ABA Vonk, D Veerhoek, S Kamminga, HJS de Grooth, JJ García-Vallejo, RJP Musters, ARJ Girbes, HM Oudemans-van Straaten, AME Spoelstra-de Man

**Affiliations:** VU University Medical Center, ICaR-VU, Intensive Care, Amsterdam, Netherlands; VU University Medical Center, ICaR-VU, Internal Medicine, Amsterdam, Netherlands; VU University Medical Center, ICaR-VU, Anesthesiology, Amsterdam, Netherlands; VU University Medical Center, ICaR-VU, Cardiothoracal Surgery, Amsterdam, Netherlands; VU University Medical Center, ICaR-VU, Clinical Perfusion, Amsterdam, Netherlands; VU University Medical Center, ICaR-VU, Molecular Cell Biology and Immunology, Amsterdam, Netherlands; VU University Medical Center, ICaR-VU, Physiology, Amsterdam, Netherlands

## Introduction

Hyperoxia is frequently encountered in the intensive care unit (ICU) and during surgical procedures such as coronary artery bypass surgery (CABG). Higher oxygen concentrations intuitively provide a salutary oxygen reserve, but hyperoxia can induce adverse effects such as systemic vasoconstriction, reduction of cardiac output, increased microcirculatory heterogeneity and increased reactive oxygen species production. Previous studies in patients undergoing CABG surgery suggest reduced myocardial damage when avoiding extreme perioperative hyperoxia (>400 mmHg). Here, we compare moderate hyperoxia to near-physiological values.

## Objectives

To investigate whether an oxygenation strategy towards lowering perioperative PaO_2_s from moderate hyperoxia to near-physiological values decreases myocardial damage and organ injury.

## Methods

In this single-center, open-label randomized-controlled trial, 50 patients scheduled for elective isolated CABG surgery were allocated to either a PaO_2_ target according to common practice (200-220 mmHg during cardiopulmonary bypass (CPB) and 130-150 mmHg in the ICU) versus a lower PaO_2_ target (130-150 mmHg and 80-100 mmHg, respectively). The primary outcome was myocardial damage (CK-MB and Troponin-T), which was determined before surgery, at ICU admission and 2, 6 and 12 hours thereafter.

## Results

**Figure 1:**
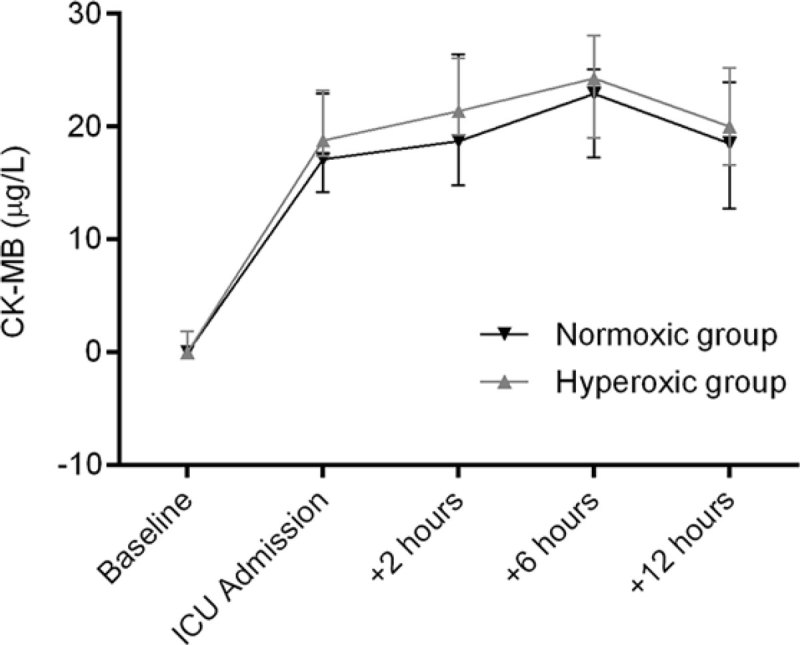
**CK-MB levels (median ± IQR)**.

Baseline and surgery characteristics were not different between groups. The mean age of patients was 66 years (SD 8) vs. 68(6), respectively. Mean duration of CPB was 105 minutes (SD 24) vs. 108(28). Weighted PaO_2_ during CPB was 220 mmHg, IQR (211-233) vs. 157 (151-162, *P*< 0.0001), respectively. In the ICU, weighted PaO_2_ was 107 (86-141) vs. 90 (84-98, *P* = 0.03). Median maximum values of CK-MB were 25.8µg/L, IQR (20.3-32.6) vs. 24.9 (18.0-31.2, *P* = 0.5) and of Troponin-T 0.35 µg/L, IQR (0.30-0.46) vs. 0.42(0.26-0.49, *P* = 0.9). Areas under the curve (AUC) of CK-MB (Figure 1) were median 23.5 µg/L/h, IQR (18.4-28.1) vs. 21.5(15.8-26.6, *P* = .35) and 0.30µg/L/h (0.25-0.44) vs. 0.39 (0.24-0.43, *P* = .81) for Troponin-T. Cardiac Index, Systemic Vascular Resistance Index, and serum lactate levels (Lactate_max_ median 2 mmol/L IQR(1.4-2.6) vs. 2.2(1.7-2.6, *P* = .52)) were similar between groups throughout the ICU period.

## Conclusions

In the present RCT, an oxygenation strategy towards near-physiological arterial oxygen tensions did not reduce myocardial damage in comparison to moderate hyperoxia. On the other hand, conservative oxygen administration did not lead to increased lactate levels.

## Grant Acknowledgment

This investigation was supported by grants from ESICM NEXT Start-Up 2014 and ZonMW

